# Should level V be included in lateral neck dissection in treating papillary thyroid carcinoma?

**DOI:** 10.1186/1477-7819-11-304

**Published:** 2013-11-25

**Authors:** Xiao-Jun Zhang, Dan Liu, De-Bin Xu, Ya-Qi Mu, Wen-Kuan Chen

**Affiliations:** 1 Department of Head and Neck Surgery, State Key Laboratory of Oncology in South China; Collaborative Innovation Center for Cancer Medicine, Sun Yat-sen University Cancer Center, 651 Dongfeng Dong Road, Guangzhou 510060, P. R. China; 2 Department of General Surgery, The Fifth People’s Hospital of Shenzhen, 47 Youyi Road, Shenzhen 518000, P. R. China; 3 Department of Thyroid Surgery, The Second Affiliated Hospital of Nanchang University, 1 Minde Road, Nanchang 330000, P.R. China; 4 Department of Oncology, The First People’s Hospital of Kaifeng, 85 Hedao Street, Kaifeng 475000, P. R. China

**Keywords:** Papillary thyroid carcinoma, Lymph node metastasis, Lateral neck dissection

## Abstract

**Background:**

The study was designed to explore the regular patterns of level V lymph node metastasis (LNM) in papillary thyroid carcinoma (PTC), and to indicate whether level V should be included in the management of lateral neck dissection when treating PTC.

**Methods:**

This retrospective study consisted of 330 patients diagnosed with PTC from January 1994 to July 2009 who underwent an operation that included therapeutic lateral neck dissection (levels II to V). The patterns of lateral neck LNM were analyzed and the relevant risk factors of level V LNM were analyzed with univariate and multivariate analysis, respectively.

**Results:**

All the patients underwent lateral neck dissection at levels II to V. The predominant site of metastasis was level III (247/330 (74.8%)), followed by level IV (233/330 (70.6%)), and level II (215/330 (65.3%)). Simultaneous multilevel involvement (level II, III, and IV) of lymphatic metastases presented in 46.1% (152/330) of the cases. Level V showed 28.8% (95/330) of nodal metastasis. Multivariate analysis showed that level V LNM was significantly associated with location (whole thyroid), gross extrathyroidal extension and simultaneous multilevel involvement (level II, III and IV). (*P* <0.05).

**Conclusions:**

Due to relatively high rate of level V involvement and its correlation with location (whole thyroid), gross extrathyroidal extension and multilevel involvement, we consider that it may be more rational to include level V in the therapeutic lateral neck dissection when treating PTC, especially for those who have any one of these three independent risk factors.

## Background

Papillary thyroid carcinoma (PTC) seems to display a peculiar characteristic in which some biological discrepancies exist between clinical and subclinical cancers. For example, although the prevalence of microcarcinoma in autopsy series has been reported up to 36% among the general population, clinical prevalence of PTC is 0.1% at the most. The patients with PTC have a relatively excellent prognosis with a 10-year overall survival rate exceeding 90%. However, cervical lymph node metastasis are found in 30 to 80% of patients when diagnosed, including occult metastases [1]. Cervical LNM in PTC has been found to be one of the most significant factors for locoregional recurrence, and it also has been regarded as the first sign of a potentially lethal outcome [2-5]. Therefore, an effective therapeutic lateral neck dissection is critical. However, overly aggressive operations such as blindly enlarging the extent of lateral neck dissection may lead to clinically important postoperative morbidities because of injury to the spinal accessory nerve or the cervical plexus, despite gross preservation of these nerves [6,7]. Consequently, it is of great clinical significance to extensively and deeply understand the regular patterns of cervical LNM, which could indicate the rational extent of lateral neck dissection in the treatment of PTC.

At present, most scholars agree that therapeutic or prophylactic central neck dissection should be routinely performed in treatment of PTC. As for the lateral neck, unfortunately, the extent of dissection remains controversial. Patients of PTC with lateral neck lymph node metastases should undergo therapeutic lateral neck dissection. Generally, the extent of therapeutic lateral neck dissection includes level II to V. However, some authors suggested that routine level V lymphadenectomy was not necessary for the treatment of patients with PTC who have clinically positive lateral neck lymph node metastases because of the relatively low metastases rate of lymph nodes in level V [8]. Another opinion is that PTC is associated with LNM in 30 to 80% of patients pathologically, while nodal recurrence in patients not receiving prophylactic dissection is reportedly less than 10%. The aim of this study was to explore the regular patterns of lateral neck LNM and suggest the rational management of lateral neck dissection in treatment of PTC: should level V be included in lateral neck dissection in the treatment of papillary thyroid carcinoma?

## Methods

We collected clinical data of 330 patients with PTC from January 1994 to Jul 2009 in Sun Yat-sen University Cancer Center. The Institutional Review Board of Sun Yat-sen University Cancer Center approved the study. All patients received initial treatment of therapeutic lateral neck dissection at level II to V for treatment of clinically positive lateral neck nodal metastases. Physical examination, thyroid ultrasonography (US) and neck computed tomography (CT) were performed for preoperative evaluation of the primary thyroid tumors and suspicious lateral neck lymph nodes, and PTC and lateral neck lymph node metastasis were both confirmed pathologically by fine-needle aspiration cytology or intraoperative frozen section. All neck dissection specimens were separated by regions for pathological analysis. The patterns of lateral neck LNM were analyzed. Also the relevant risk factors of level V LNM were analyzed, with univariate and multivariate analysis, respectively. The ‘respectively’ refers to ‘the relevant risk factors of level V LNM’ only. All of the 330 patients received thyroidectomy (total or lobectomy plus isthmusectomy) together with central neck dissection and therapeutic lateral neck dissection (levels II to V). Indications for total thyroidectomy are mainly based on the conventionally high-risk factors, including age younger than 15 years or older than 45 years, radiation history, known distant metastases, cervical lymph node metastases, extrathyroidal extension, tumor greater than 4 cm in diameter, bilateral nodularity, or aggressive variants like tall cell, columnar cell or diffuse sclerosing variant. Patients without above high-risk factors received lobectomy plus isthmusectomy. Of those patients, 29 patients underwent bilateral lateral neck dissection. All neck dissection specimens were recorded according to regions, but only the ipsilateral specimens were analyzed in this study when bilateral lateral neck dissection was performed.

Within a median follow-up of 8.9 years (4.0 to 18.0 years), 12.1% (40/330) of this patient cohort has tumor recurrences; up to 75% (30/40) of these recurrences were seen in cervical lymph nodes, and 25% (10/40) were seen in thyroid remnants. Distant metastases were seen in 3.3% (11/330), most often (72.7%) in lungs. 4.2% (14/330) of cases resulted in cancer-related death. The changes did not alter intended meaning of the previous sentence.

Statistical analyses were performed using SPSS ver. 13.0 (SPSS Inc., Chicago, IL, USA). Univariate analyses for the associations between level V lymph node metastases and clinicopathologic factors of thyroid carcinoma were performed using Pearson Chi-square test. Multivariate analysis was performed using Binary logistic regression analysis. A *P* value of less than 0.05 was considered statistically significant.

## Results

Of these 330 patients with PTC that involved lateral neck lymph node metastases, 140 were male and 190 were female. The median age was 35.0 years (range, 8 to 75 years) and there were 243 (73.6%) patients who were younger than 45 years. The number of patients with solitary and multifocal lesion of primary tumor were 248 (75.2%) and 82 (24.8%), respectively (Table 1).

**Table 1:** Patients’ demographics

**Variable**		**Value**	**Ratio (%)**
Gender	Male	140	42.4
	Female	190	57.6
Age (years)	<45	243	73.6
	≥45	87	26.4
Location^a^	left lobe	120	36.4
	right lobe	152	46.1
	whole thyroid	58	17.5
Lesion	solitary lesion	248	75.2
	multifocal lesion	82	24.8
Intact capsule^b^	Yes	292	88.5
	No	38	11.5
Gross Ex	Yes	39	11.8
	No	291	88.2
Tumor size (cm)	≤1	56	17.0
	>1, ≤ 2	140	42.4
	>2, ≤ 4	117	35.5
	>4	17	5.2
Local recurrences	Yes	40	12.1
	No	290	87.9
Distant metastases	Yes	11	3.3
	No	319	96.7
Death	Yes	14	4.2
	No	316	95.8

^a^The site of primary papillary thyroid carcinoma (PTC).

^b^Intact capsule means that tumor have not extended through its capsule.

Gross Ex, gross extrathyroidal extension.

As to the lateral neck lymph node metastases, the predominant site of metastasis was level III (247/330 (74.8%)), followed by level IV (233/330 (70.6%)), and level II (215/330 (65.3%)). Level V showed 28.8% (95/330) of nodal metastasis. Single level involvement was seen in 68 (20.6%) cases. Simultaneous multilevel involvement (level II, III, and IV) of lymphatic metastases was seen in 152 (46.1%) cases. Univariate analysis of risk factors related to level V LNM demonstrated that, level V LNM was associated with location, gross extrathyroidal extension and simultaneous multilevel involvement (level II, III, and IV) of lymphatic metastases (*P* <0.05, Table 2). In multivariate analysis, level V LNM was significantly associated with location (whole thyroid), gross extrathyroidal extension and simultaneous multilevel involvement (level II, III and IV) (*P* <0.05, Table 3).

**Table 2:** Univariate analysis of factors related to level V lymph node metastasis (LNM) in 330 patients with papillary thyroid carcinoma (PTC)

**Variable**		**Cases**	**LNM or**	**Not**	***P*** **value**
Age	<45 yr	243	66	177	0.275
	≥45 yr	87	29	58	
Gender	Male	140	47	93	0.099
	Female	190	48	142	
Location^a^	Left lobe	120	29	91	0.000
	Right lobe	152	37	115	
	Whole thyroid	58	29	29	
Lesion	Solitary lesion	248	67	181	0.216
	Multifocal lesion	82	28	54	
Intact capsule^b^	Yes	292	81	211	0.244
	No	38	14	24	
Gross Ex	Yes	39	17	22	0.030
	No	291	78	213	
Tumor size (cm)	≤1	56	12	44	0.170
	1 ≤2	140	44	96	
	2 ≤4	117	31	86	
	>4	17	8	9	
Simultaneous	Yes	152	53	99	0.024
metastases^c^	No	178	42	136	

^a^The site of primary PTC.

^b^Intact capsule means that tumor have not extended through its capsule.

^c^Simultaneous metastases including level II, III and IV.

Gross Ex, gross extrathyroidal extension.

**Table 3:** Multivariate analysis of factors related to level V lymph node metastasis (LNM) in 330 patients with papillary thyroid carcinoma (PTC)

**Variable**	***P*** **value**	**Risk ratios**	**95.0% C.I.**
Location^a^	0.002	0.591	0.421 to 0.829
Gross Ex^b^	0.049	2.014	1.004 to 4.038
Simultaneous metastases^c^	0.004	2.074	1.271 to 3.385

^a^The site of primary PTC.

^b^Gross extrathyroidal extension.

^c^Simultaneous metastases including level II, III and IV.

## Discussion

As an excellent prognosis for patients with PTC is possible, increasingly, doctors and patients have begun to pursue the second aim: decreasing recurrence and chance of second operation and improving the patients’ quality of life. Early cervical lymph node metastasis is one of the most remarkable features in PTC. Therefore, it is essential to effectively manage the metastasizing lateral lymph nodes.

There are various options in the extent of therapeutic lateral neck dissection in PTC. Whether or not level V should be included in the lateral neck dissection continues to be debated [9,10].

In this study, the predominant site of metastasis was level III (247/330 (74.8%)), followed by level IV (233/330 (70.6%)), and level II (215/330 (65.3%)). The incidence of simultaneous multilevel involvement of lymphatic metastases was as high as 46.1%, which was in accordance with the data reported in literature [11]. Hence, at least level II through IV should be included in the therapeutic lateral neck dissection if the lateral neck lymph nodes were positive.

Young Chang Lim *et al*. [8] reported that occult metastases in level V were observed in 11/70 (16%) patients, and it was not appropriate to routinely perform Level V lymphadenectomy. But Kupferman *et al*. [12] believed that routine dissection of the level V lymph nodes was necessary in the therapeutic lateral neck dissection. In this study, level V showed 28.8% (95/330) of nodal metastasis and agreed with the incidence of approximately 16.7 to 53% reported in literature [11-16]. Multivariate analysis indicated that location (whole thyroid), gross extrathyroidal extension and simultaneous multilevel involvement (level II, III, and IV) of lymphatic metastases were the independent risk factors of level V LNM (Table 3), which was similar to the results reported in the literature [12,13,17]. In a word, due to both the relatively high incidence (28.8%) of level V LNM and incidence (46.1%) of simultaneous multilevel involvement (level II, III, and IV) of lymphatic metastases in this study, we consider that it may be more rational to include level V in the therapeutic lateral neck dissection, especially when primary PTC is located in the whole thyroid, or when gross extrathyroidal extension or multilevel involvement are present.

Considering that this is a retrospective study and information in the pathological report is limited, we failed to separate the analysis for levels Va and Vb. We will analyze the levels in our further studies. Nevertheless, we present a larger sample size in this study and demonstrate a reliable result in the management of level V when therapeutic lateral neck dissection was performed for the treatment of PTC.

## Conclusions

In summary, due to relatively high rate of level V involvement and its correlation with location (whole thyroid), gross extrathyroidal extension and multilevel involvement, we consider that it may be more rational to include level V in the therapeutic lateral neck dissection when treating PTC, especially for those who have any one of these three independent risk factors.

## Abbreviations

CT: Computed tomography; LNM: Lymph node metastasis; PTC: Papillary thyroid carcinoma; US: Ultrasonography.

## Competing interests

The authors declare that they have no competing interests.

## Authors’ contributions

WC participated in the conception and design of the study and carried out the surgery. XZ and DL participated in the design of the study, data analysis and interpretation and drafted the manuscript. DX and YM participated in the collection of the data. All authors read and approved the final manuscript.
